# Patients’ and tutors’ evaluations of medicine students’ consultations in general practice/family medicine in Coimbra

**DOI:** 10.1186/s12909-020-02042-3

**Published:** 2020-05-06

**Authors:** Luiz Miguel Santiago, Inês Silva, José Augusto Simões

**Affiliations:** Faculty of Medicine of the University of Coimbra, General Practice and Family Medicine University Clinic of the University of Coimbra, Coimbra, Portugal

**Keywords:** Clinical competence, Education, Family medicine, General practice, Undergraduate, Patient care planning

## Abstract

**Background:**

Undergraduate teaching of General Practice/Family Medicine (GP/FM) must ensure students acquire the necessary competencies and skills to perform an adequate GP/FM consultation with adequate annotations (the SOAP model) and classifications. So aimed to study and to correlate students’ evaluation by tutors and patients in specific consultations in the formal practical evaluation of GP/FM Curricular Unit of the Integrated Masters on Medicine at the Faculty of Medicine of the University of Coimbra (IMM-FMUC) in the academic years of 2017–2018 and 2018–2019.

**Methods:**

Observational study of the 2017–2018 and 2018–2019 academic years of the assessment grids for tutor’s evaluation of SOAP performance and fluency in consultation and for patient’s evaluation of the student ‘performance, in the convenience sample of those who chose to be so evaluated.

**Results:**

We studied a population of 435 (67,7%) out of a universe of 646 students, 125 (28,7%) males, ns by sex and academic year who performed this evaluation. In a mark up to 20 from tutors, difference was found for Plan (P) mark, higher in 2018–2019 (18,38 ± 2,18*vs*18,54 ± 2,11, *p* = 0,005) of the SOAP methodology evaluation. Patients’ evaluation was not different 19,34 ± 1,70*vs*19,35 ± 1,40, p = 0,091. A positive significant correlation was found between tutors and patients marks (ρ = 0,278; *p* < 0,001), as well as between tutor mark and final mark (ρ = 0,958; p < 0,001) and patient and final marks (ρ = 0,465; p < 0,001). Final marks were not different in both years, 18,61 ± 1,38*vs*18,78 ± 1,15, *p* = 0,158.

**Conclusions:**

This innovative model of evaluation of student’s performance in medical appointment, showed a significant positive moderate correlation between patients’ and tutors’ marks in the setting of GP/FM at the IMM-FMUC, and was not different between years. Yearly evaluation must be continued.

## Practice points

1. General Practice/Family Medicine (GP/FM) consultations are ever more about people’s fears, doubts and expectations as well as about the correct and precise translation of the Medical knowledge, so students must be prepared to deal with them.
2. Undergraduate teaching of (GP/FM) must ensure students acquire the necessary competencies and skills to perform an adequate consultation.
3. The practice of the SOAP model for consultation is essential for a good result and out-come from an appointment, in the light of Patient Centered Medicine.
4. The assessment and evaluation by pre-specified grids with feed-back is important.
  Patients are important student’s feed-back for the analyses of skills and communication in a consultation.
5. Positive significant correlations are found between tutors and patients marks

## Highlights

- Consultation is the work setting for future GPs/FDs, mostly out of the hospital.
- The ability of medical students to communicate and make relevant clinical notes and classifications according to the SOAP model, while keeping the fluency of the consultation, has proved to be pivotal to ensure favorable outcomes during medical consultations.
- No significant differences were found in both tutor’s and patient’s final scores in a two-sequential-academic-year period.
- This approach to evaluate the skills and capabilities of medical students shows promising results, but further studies should be developed.

## Background

Teaching General Practice/Family Medicine (GP/FM) at the undergraduate level is a paramount task to make future General Practitioners/Family Doctors (GP/FDs) meet the goals set by the WONCA-EURACT Definition [1].

The main competencies can be taught but theory will not, surely, give students the skills they need to fulfill the task of performing an adequate consultation: communicating empathically, listening and asking, performing adequate medical exam, assessing, preparing a plan and explaining it and at the same time, righting it clearly.

In fact, consultation is the work setting of the future GP/FDs, mostly out of hospital [1].

Patients are, in general, favorable to the presence of medical students and there seems to be much to gain by students, patients and tutors with such practical approach [2–6].

The “*Blueprint for an Undergraduate Primary Care Curriculum*” underpins care management with longitudinally, generalist, central responsibility for managing care, therapeutic alliance/communication, approach to acute care, approach to chronic care, wellness and prevention, mental and behavioral health, interprofessional training, systems improvement and population health are key issues to be taught to future GP/FDs, so fulfilling what the EURACT definition states [1, 7].

As teachers and tutors must make assessments and evaluate students, so should patients evaluate students in consultation, such evaluation being intended as a self-learning tool for students and tutors [6]. To do so a systematic organized recoil of information is mandatory beginning by the reason for consultation the S from Subjetive, including signs and symptoms the patient is suffering of and the fears and expectations as well as the answers to doctor questions, continuing in O from Objective introducing the signs in physical exam and even the psychological viewing of the patient and the results of tests, following by A from Assessment, the doctor diagnostics and finishing in P form Plan, where the prescribed medications, exams and non-pharmacologic therapies as well as other measures of enablement and empowerment must be written for future memory [6, 8]. This procedure implies clinical ordered information to be easily look for in the future, allowing epidemiologic studies to be performed, should that information be classified according to ICPC2, the aanotations and classifications constituting the clinical registries [6, 8, 9].

At the Faculty of Medicine of the University of Coimbra (FMUC), since 2017–2018, students begin contact with GP/FM in the fifth of a 6 years Integrated Master’s in Medicine (MIM), attending 30 h on-class inter-active sessions with theory, role-play and video watching and discussions. They also attend a minimum of 8 hours in a Primary Care Family Medicine unit with a trained tutor, to observe and practice, being voluntarily evaluated near the end of such period by performing a tutor’s selected consultation, of an appropriate degree of difficulty. Matters like Subjective, Objective, Assessment and Plan, the SOAP methodology, International Classification for Primary Care2 (ICPC2), Patient Centered Medicine, Medical Empathy and empa thic opportunities, Enablement and empowerment, Preventive Medicine, Multimorbidity, Polypharmacy, Psychological problems, Ageing problems and Primary Care were presented and discussed in-class with students [8–19]. Particular interest was put on Patient Centered Medicine and empathy as a way to focus our students on the person suffering and not only on diseases [7, 11, 20, 21].. The Dean of the FMUC issued a consent form approving the Curricular Unit Form once the FMUC Pedagogic Council gave its approval to the proposed scheme of teaching GP/FM from the year 2017–2018 onwards.

Until 2017 the work-out for the curricular discipline practical examination was a mandatory study of a Family, filling in a free report to be assessed, followed by a voluntary oral presentation with no benefit for better mark for the student.

Since GP/FM in the Portuguese National Health Service (PNHS) is practiced out of hospitals in small units with doctors, nurses and secretaries, it was intended that medical students practiced in such a setting questioning, examining, diagnosing and making plans for exams, non-pharmacological and pharmacological prescriptions as well as explaining it efficiently to patients, with proper e.registrations. In the PNHS, an informatics program is used, so students also learned about it through their tutors. Tutors were experienced GP/FD specialists that attended at least three two hours sessions on what was intended to be explained and how it was to be measured.

A previous study, in a very different medical education context in Portugal, found a neutral non-significant correlation between patients’ and tutors’ evaluation of the consultations in GP/FM in 4th grade students. It was hypothesized that the evaluation of the consultation by the patient could be an interesting tool to measure practical student skills when compared with the tutor’s evaluation, at the same time serving as an educational feed-back tool [6]. In fact Patient Centered Medicine and empathy are core issues to develop patient-doctor relationship which students can ignore when merely pursuing a disease centered approach [6–11].

A 2010 paper found that teachers’ scores were in accordance with patients’ scores [22] and a paper in Portugal found a neutral and non-significant correlation between the valuation mark by the tutor and the patient’s grade [6]. This means that the preparation of our students for practical clinical life must be an object of concern by Medical Schools.

### Objective

To study and to correlate students’ evaluation by tutors and patients in specific consultations in the formal practical evaluation of GP/FM Curricular Unit of the MIM at the FMUC in the academic years of 2017–2018 and 2018–2019 looking for differences.

## Methods

Observational study of the results in two consecutive academic years, 2017–2018 and 218–2019, studying the marks of those students that chose the consultation evaluation as the practical exam in a convenience sample.

Grids were developed to get a numerical objective score of a subjective assessment to evaluate the skills and the accomplishment of the SOAP methodology as well as the annotations and classifications made by 5th year MIM-FMUC students. SOAP methodology stands for S-Subjective, the reason for consultation, O-Objective or what is collected in consultation, A-Assessment, what are the diagnosis in consultation and P-Plan, what is going to the proposed to the patient to be made. They were intended to analyze and to grade particular aspects of each part of the SOAP model as well as the fluency of the consultation by the tutor and its evaluation by the patient. These issues were firstly discussed with the student’s committee, showed and discussed with students at the first and introductive class and the grids and the practical issues to be observed were published in the student’s FMUC intra-net.

Prior evaluation assessments took place in the tutor/student communication but those were not brought into evaluation. In the course of the consultation students could scales to study the individual and its family.

Clinical tutors, GP/FDs with experience and knowledge, were previously trained on how to do the task of teaching, demonstrating and evaluating in a two hour session at the beginning of September and February, each academic year, by performing and matching role-play and video consultations and by trios exercises in which they had to perform the task of being a doctor, being a patient or a tutor. Tutors were also instructed on the difficulty of each case to present giving special attention to non-scheduled consultations.

The consultation methodology SOAP marks were calculated according to Tables 1 and 2. Marks were calculated using the tutor’s mark and the patient’s mark. Those marks were the numerical evaluation of several aspects in S, O, A and P, as well as the evaluation of the fluency of the consultation by the experienced tutor. Attending of the grade of Medical knowledge, for SOAP, S represented 60%, O 15%; A 5%; and P 20% of the mark, which accounted for 60% of the tutor’s mark the rest coming from the fluency mark. For the final mark of the evaluation in 2017–2018 tutor’s mark accounted for 60%, the patient’s evaluation accounting for 40% and in 2018–2019 tutor’s mark accounted for 80% and the patient’s one for 20%, according to a suggestion of the board of the Faculty of Medicine of the University of Coimbra. Table 1 shows the tutor’s SOAP and fluency evaluation tables, and also how several observed aspects were to be graded.

**Table 1:** Tutor’s SOAP and fluidity evaluation tables

Student’s characteristics to be observed and marked	No (0)	Yes, no lapses (3)	Yes with lapses (2)
1Student presents himself			
2 Reason for encounter in the eyes of the patient			
3 The reason for encounter is clear for both student and patient			
4 There are clear annotations of what the patient said			
5 There are annotations of the physical exam according to patient’s complaints			
6 There is an evaluation with explanation of the problem or problems to deal with			
7 There is a general explanation of the plan			
8 There is a clear register of the information provided during the consultation			
Characteristics of the consultation fluence	Total (4)	Intermediate (2)	Low (1)
Security in the consultation process	□	□	□
Flowing from S to O to A to P (SOAP)	□	□	□
Technical rigor (language and gestures)	□	□	□
Communication	□	□	□

Note: 5th year: S = 1 + 2 + 3 + 4 = 60%; O = 5 = 15%; A = 6 = 5%; *P* = 7 + 8 = 20% of the mark

Fluidity as the sum of marks

**Table 2:** Patient’s evaluation

Statement	Answer			
	None (1)	Little (2)	Much (3)	Total (4)
1. I could state my reason(s) for this consultation.	□	□	□	□
2. A physical exam was made because of my complaints	□	□	□	□
3. The reason for my complaints was explained to me	□	□	□	□
4. I was suggested what to do to get better.	□	□	□	□
5. I think the student understood my problems	□	□	□	□
6. I understood the information’s I was given.	□	□	□	□
7. I enjoyed my clinical appointment.	□	□	□	□

Note: Mark as the sum of answers

The patient’s evaluation was given to the tutor by the patient that had previously given consent to this task, after answering to the questions in Table 2, so no data on who answered exists. The patient’s mark was calculated as the sum of answers, Table 2.

All data were known by the student at the end of the evaluation so that a feedback session could be made with the tutor. It was mandatory for students and tutors to sign up the paper marks at the end of evaluation. Students were given a second opportunity of consultation should they wish it, final mark being the last one.

As the studied data were analyzed anonymously, and were public no consent to participate was obtained, according to the approved protocol by the Faculty of Medicine of the University of Coimbra.

At the end of each academic year, all the information was gathered from the assessment grids in order to be studied for differences between semester, academic year and gender by descriptive and inferential parametric statistics: for nominal data we used the χ2 test and for continuous non-normal and the ordinal data the Mann-Whitney U test. Spearman correlation between patients, tutors’ and final marks were performed using IBM SPSS statistics 24. We defined *p* < 0,001 for difference.

## Results

Of a Universe of 646 (322 in 2017–2018), a population of 435 (67,3% of the total) was studied 28,7% being male. We could not know the sex of two students (Table 3).

**Table 3:** Population and sample according to academic year and sex

		Academic Year		Total
		2017–2018	2018–2019	
Sex				
Feminine	n	219	231	450
	%	68,0%	71,3%	69,7%
Masculine	n	103	93	196
	%	32,0%	28,7%	30,3%
Total	n	322	324	646
	%	100,0%	100,0%	100,0%
Population (*)				
Sex				
Feminine	n	154	156	310
	%	71,0%	71,6%	71,3%
Masculine	n	63	62	125
	%	29,0%	28,4%	28,7%
Total	n	217	218	435
	%	100,0%	100,0%	100,0%

(*)χ^2^ (Exact Fisher test) p = 0,072

According to Table 4, in a non-normal mark up to 20 distribution and comparing by academic year, no significant differences were found although higher values were found for the academic year of 2018–2019. SOAP Plan component was significantly better marked in the 2018–2019 academic year.

**Table 4:** S, O, A, P, SOAP, Fluency, Tutor, Patient at final marks in 2018 and 2019 academic years

	Academic year	N	Mean	Standard-deviation	p
S mark	2017–2018	219	19,20	1,36	0,095
	2018–2019	218	19,53	1,03	
O mark	2017–2018	219	18,81	2,56	0,350
	2018–2019	218	19,17	2,20	
A mark	2017–2018	218	17,95	3,08	0,432
	2018–2019	218	18,26	2,94	
P mark	2017–2018	218	18,38	2,18	0,005
	2018–2019	218	18,59	2,11	
Tutor’s SOAP mark	2017–2018	219	18,90	1,42	0,236
	2018–2019	218	19,09	1,21	
Tutor’s Fluency mark	2017–2018	219	16,96	3,29	0,774
	2018–2019	218	17,44	2,30	
Tutor’s mark	2017–2018	219	18,12	1,91	0,042
	2018–2019	218	18,60	1,34	
Patient’s mark	2017–2018	219	19,35	1,41	0,051
	2018–2019	218	19,53	1,15	
Final mark	2017–2018	219	18,61	1,38	0,473
	2018–2019	218	18,78	1,15	

In a mark up to 20 and comparing by sex no significant differences were found according to Table 5.

**Table 5:** S, O, A, P, SOAP, Fluency, Tutor, Patient at final marks by sex

	Sex	N	Mean	Standard-deviation	p
S mark	Feminine	312	19,39	1,21	0,323
	Masculine	125	19,31	1,24	
O mark	Feminine	312	18,93	2,45	0,408
	Masculine	125	19,15	2,24	
A mark	Feminine	311	18,16	2,99	0,735
	Masculine	125	17,97	3,08	
P mark	Feminine	311	18,46	2,20	0,741
	Masculine	125	18,56	2,00	
SOAP mark	Feminine	312	18,98	1,36	0,877
	Masculine	125	19,02	1,22	
Fluency mark	Feminine	312	17,30	2,84	0,169
	Masculine	125	16,95	2,84	
Tutor’s mark	Feminine	312	18,39	1,71	0,194
	Masculine	125	18,27	1,55	
Patient mark	Feminine	312	19,50	1,19	0,352
	Masculine	125	19,29	1,50	
Final mark	Feminine	312	18,73	1,27	0,192
	Masculine	125	18,60	1,26	

A strong positive and significant correlation was found between “Tutors' mark” and “Final mark” evaluations (ρ = 0,958, *p* < 0,001), a moderate and significant one between “Patient's mark and Final Mark” (ρ = 0,465, p < 0,001) and a week but significant on between “Tutor's mark and Patient's mark” (ρ = 0,278, p < 0,001).

## Discussion

In a predominantly female population, students’ evaluation performance in consultation is not different by academic year or gender.

Out of 646 students, 435 (67,3%) chose the consultation evaluation. Even though not statistically different, students in the 2018–2019 academic year and females tend to be scored higher. We acknowledge that a bias of performance can exist once students can perform consultation like they were taught just for the sake of being well evaluated but without a clear and incisive belief on such a model of consultation centered on the patient and not only on the disease.

The calculated correlations mean that patients and tutors tend to evaluate similarly to another paper [22] but differently from a previous Portuguese study [6]. If in the case of the former study there are similar results with different evaluation grids, for the latter more studies at the FMUC and in other Faculties of Medicine in Portugal seem necessary.

We aimed to measure in the setting of a GP/FM consultation, the performance of the SOAP model, the registries, the practice of Patient Centered Medicine, the physical exam focused on the patient’s complaints performing the necessary physical exam according to ethics, the diagnosis capacity, the communication skills, the ability of negotiating, enabling and empowering a patient and the annotations and ICPC2 classifications made and we found no differences between the two studied populations comparing 2017–2018 to 2018–2019, which was our aim. A week but significant difference between “Tutor’s mark and Patient’s mark” being found is a matter deserving future studies. It is possible that patient’s satisfaction is different from the one of tutors. The scientific translational medical knowledge was not specifically evaluated, an OSCE being needed to fulfill such a task [23–25].

Students had access to all the assessments. All consultation’s participants signed forms were personally handed by the student so serving as feed-back information.

We believe that this model is suited to measure what it is intended to measure. In fact a consultation as we teach it in the Patient-Centered Medicine Methodology must include the focus on empowerment and answer to patient’s questions, the illness, so being a complete exercise students were not yet exposed to up to the 5th year at FMUC.

Nevertheless:

1. Listening to students, eventually by a self-administered questionnaire about the consultation;
2. Investing more time in-practice with more consultations and making follow-up consultations for previous patients, averaging the mark as the mean of at least three consultations, are important issues to deal with in the future.

## Conclusions

A significant positive moderate correlation exists between patients’ and tutors’ marks when evaluating the practice of consultations in the setting of General Practice/Family Medicine at the Faculty of Medicine of the University of Coimbra, patients being more satisfied with student’s consultations than tutors.

This model deserves further development and future studies, namely for the kind of patients and pathologies the students are exposed to and the kind of registries they do in consultation..

## Data Availability

All data will become available if requested.

## Abbreviations

GP/FM: General Practice/Family Medicine
MIM: Integrated Master’s degree in Medicine
FMUC: University of Medicine of the University of Coimbra,
SOAP: Subjective, Objective, Assessement, Plan
WONCA: *World Organization of National Colleges, Academies and Academic Associations of General Practitioners/Family Physicians*
EURACT: European Academy of Teachers in General Practice Family Medicine
GP/FD: General Practitioner Family Doctor
ICPC2: International Classification for Primary Care2
OSCE: Oriented Structured Clinical Examination
